# Current Concepts in Pediatric Cervical Spine Trauma

**DOI:** 10.2174/1874325001711010346

**Published:** 2017-04-28

**Authors:** Kunal Shah, Agnivesh Tikoo, Manish K. Kothari, Abhay Nene

**Affiliations:** Wockhardt Hospitals, 1877, Dr. Anand Rao Nair Road, Mumbai Central (E), Mumbai- 400 011, India

**Keywords:** Cervical, Diagnosis, Management, Pediatric, Synchondrosis, Trauma

## Abstract

**Background:**

Pediatric spinal trauma is rare and challenging entity. Although cervical spine is commonly affected, it is often missed on routine imaging investigations. Therefore better understanding of growing spine and its patho-physiology is crucial.

**Methods:**

Articles related to pediatric cervical trauma were searched on Pubmed and other online research data banks. We have summarized unique anatomy of pediatric spine, investigations followed by common injury patterns, their diagnostic challenges and management.

**Results:**

Immature spine follows typical injury patterns, so thorough knowledge of its presentation should be known. Primary physicians should be able to perform initial assessment based on clinical examination and investigations for early diagnosis. High index of suspicion and strategic approach leads to early diagnosis and prevents further morbidity and mortality.

**Conclusion:**

Spinal injuries in children are rare and typical. They are often missed and can have fatal consequences. Thorough understanding of pediatric anatomy and injury patterns helps in early diagnosis.

## INTRODUCTION

Pediatric spine fractures are rare and accounts for 1-2% of all pediatric traumas. Cervical spine trauma is the commonest spinal injuries in children [1]. The immature growing cervical spine has unique anatomic, physiologic and biomechanical features. This leads to typical injury patterns seen in this age group. Thorough initial assessment based on history, physical examination and investigations is mandatory for early and accurate diagnosis. Spinal injury in young children can have fatal consequences even with minimal structural damage because of high flexibility of spine [2]. Permanent neurological damage is reported in upto 60% of patients with mortality as high as 40% [1, 3, 4]. Therefore high index of suspicion and systematic approach leads to appropriate diagnosis and subsequent treatment. This largely limits morbidity and mortality in these patients. 

### Unique Anatomy of Pediatric Cervical Spine

Children with age less than 8 years have relatively large head compared to a smaller trunk. This leads to upper cervical spine (C3 level) to act as a fulcrum in sudden acceleration /deceleration causing higher upper cervical spine injuries [5].

There is high amount of ligamentous laxity in spinal column. The pediatric spinal column can stretch upto 5 cm whereas the spinal cord can stretch only upto 5 mm. This can cause a spinal cord injury previously called as SCIWORA (spinal cord injury without radiological abnormality) [2]. The facet joints in children are more horizontally placed as compared to adults. The vertebral bodies are flatter and have greater compliance, increasing the chances of ligamentous injury. Other differences include less developed neck musculature and underdeveloped uncinate process. The open synchondrosis are also susceptible to trauma and are relatively difficult to diagnose with routine imaging [6].

### Initial Assessment

History taking and initial assessment is challenging in pediatric trauma. Numerous factors like patient/relatives anxiety, lack of clinical experience in emergency room, unconscious patient may make the assessment challenging. Treating doctors in emergency should be highly suspicious about injury patterns in children and should take quick decisions on further investigations and management [7]. A child suspected of spine injury should be appropriately immobilized during transport. Specialized backboard is recommended to prevent neck flexion because of a larger head. Bump beneath upper thoracic spine or cutout in board for head to transport child with spine in neutral alignment is usually used [8].

### Radiographs

**Fig. (2):**
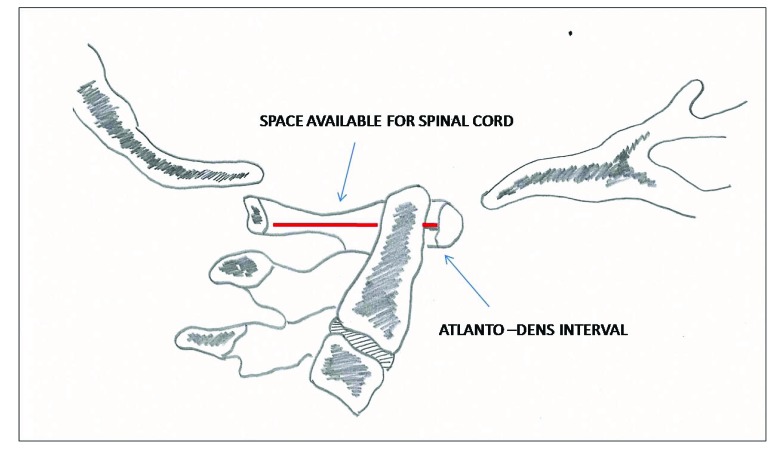
Space Available for cord (SAC) and Atlanto Dens Interval (ADI).

Plain anteroposterior, lateral and open-mouth x rays views can be used to assess and clear the cervical spine in cooperative children with a normal physical examination and a low-energy trauma [9]. Flexion-extension views should not be done in acute trauma setting; they are used to diagnose late spinal instability [10]. The anterior cervical line, posterior cervical line and spinolaminar line should be carefully seen for disruption to pick up subtle ligamentous injuries. Wackenheims line passes along the posterior arch of clivus and intersects the posterior third of dens. Powers ratio is measured by dividing the distance between basion and posterior arch C1 to distance between opisthion and posterior arc C1 [4]. McRae line, McGregor line and Chamberlain line as shown in Fig. (**1**) helps to determine basilar impression. Normal radiographic parameters in the pediatric cervical spine should be borne in mind to avoid misdiagnosis. This includes increased atlanto-dens interval (Fig. **2**) normally less than 5 mm in children, pseudosubluxation of C2 on C3 less than 4 mm, widened retropharyngeal space more than 6 mm at C2 and greater than 22 mm at C6, neurocentral synchondrosis closes by 7 years of age, loss of cervical lordosis and anterior wedging of vertebral bodies [2, 11, 12].

### Conventional Tomography (CT) Scan

Conventional radiography is not useful in uncooperative and unconscious patients. In these situations, CT scans are highly helpful as they are done quickly without any sedation or anesthesia. Specialized CT protocols are set in many centres to limit radiation exposure. Generally a kilovoltage of less than 120 kVp and tube current ranging from 60 to 120 mAs is used depending on the age and size of the child [4, 13]. CT scan provides excellent view of bony architecture, however pediatric spine is mainly cartilaginous and ligamentous injury are common and thus its routine use is debated. It is advisable to use limited CT in the area of injury as seen in plain roentgenograms. Normal measurements of upper cervical spine on CT as shown in various reports are powers ratio less than 0.9, atlanto-dental interval less than 2.6 mm, atlanto-occipital interval less than 2.5 mm, atlantoaxial interval less than 3.9 mm, basion dens interval with ossification less than 9.5 mm and without ossification less than 11.6 mm [14, 15].

### Magnetic Resonance Imaging (MRI) Scan

MRI is highly useful to assess ligamentous and cartilaginous injuries especially when plain roentgenograms and CT scan show equivocal results. It is also useful to evaluate posttraumatic disc herniation and cord hemorrhage or edema. Its utility in clearing cervical spine in a obtunded child admitted for more than 3 days with equivocal radiographs and neurological involvement is well proven [16, 17]. STIR images are particularly useful in evaluating capsular injuries and fluid accumulation in atlanotoccipital joint and atlanto axial joints [18].

## INJURY PATTERNS

### Occipital Condyle Fractures

These are rare injuries in children. Surgeons should have high index of suspicion for diagnosing it. A child presents with head injury, altered consciousness, lower cranial nerve deficit and persistent neck pain. A CT scan is always useful to know the extent of injury [19]. Occipital condyle fractures were classified by Anderson into three types. Type 1 is impaction fracture, type 2 is basilar skull fracture with condylar extension and type 3 as alar ligament avulsion fracture. Treatment includes halo immobilsation or occipito cervical arthrodesis. Type 3 injuries are usually unstable and may require surgical intervention [20].

### Atlanto-Occipital Dislocations

These are highly fatal injuries, should be looked for in children with high velocity trauma having head and facial injuries. Primary stabilizers of atlanto occipital joint are alar ligaments, tectorial membrane, anterior longitudinal ligaments and nuchal ligament. Flat occipital condyles with shallow C1 upper articular facets predispose for a translational injuries in children [21]. Clinically child presents with variable sensory -motor deficit, cranial nerve palsies,vomiting, headache, respiratory compromise and brain stem dysfunction due to vertebral artery compromise. MRI can best delineate injury to alar ligament and tectorial membrane [22]. Traction is strictly contraindicated and nonoperative management have limited role. Treatment includes occiput to C1 fusion if C1/2 articulation is preserved. However many authors prefer to fuse up to C2 for the reason that thin C1 arch may be inadequate to provide good fusion and long term stability [23].

### Atlas Fractures

Although atlas fractures are uncommon in children, they tend to occur through synchondrosis. Atlas is made of three ossification centres. The posterior synchondrosis fuses at around 3 years and the neurocentral synchondrosis fuse by 7 years of age [24]. It is best diagnosed on CT scan.

Combined lateral overhang of lateral masses greater than 6.9 mm is diagnostic for transverse ligament disruption. Treatment includes halo traction to widen lateral masses followed by halo immobilization [2, 25].

### Atlantoaxial Rotatory Subluxation

Common causes of atlantoaxial rotatory subluxation are trauma and infection other rare causes include congenital and iatrogenic. Infectious etiology also known as Grisel syndrome which occurs commonly after upper respiratory infection, but can also occur after tonsillectomy or retropharyngeal abscess [26]. The patient presents with pain, restricted range of motion and torticollis. Typical cock -robin position is described with head rotated to one side with some lateral flexion [27]. Fielding classified it into four types. Type 1 is a unilateral facet dislocation. Type 2 includes unilateral facet dislocation with anterior displacement of 3-5 mm which may signify transverse ligament rupture. Type 3 includes bilateral facet dislocation with anterior displacement. Type 4 includes posterior displacement of atlas. Type 3 and type 4 are associated with high neurologic injury and death [28]. It is best diagnosed with help of dynamic CT scan. Treatment of early rotatory subluxation includes anti-inflammatory medications, muscle relaxants and physical therapy. Patients with persistent atlantoaxial rotatory subluxation for more than 3-4 week are treated with halo traction. If successful reduction is achieved then halo immobilization is done. If it does not reduce then atlantoaxial fusion is performed [27].

### Odontoid Fractures

Odontoid fractures are commonly seen in children and usually occur through synchondrosis.

The synchondrosis at the base of odontoid fuses between 3 - 6 years of age. Peak incidence is around 4 years. Most commonly it is a Salter type 1 epiphyseal injury [29].

The mechanism of trauma in children is hyperflexion injury unlike in adults where it is caused by hyperextension injuries. Roentgenograms show anterior displacement of odontoid in lateral view which is best delineated on a CT scan. Thick anterior periosteal sleeve usually prevents gross displacement of dens. Often the odontoid may reduce after injury making the diagnosis difficult [30]. In that case a MRI may be useful because it can show edema around the epiphyses suggesting injury. Halo immobilization of neck for 6-8 weeks in extension or reduction position is the treatment usually advised [31].

### Os Odontoideum

Various authors have proposed multiple theories about origin of Os Odontoideum stating it to be of congenital origin or unrecognized odontoid fracture. Most commonly it is asymptomatic; however it can present with instability, transient neurology or myelopathic signs. Open mouth views shows a round ossicle with sclerotic margins separated from odontoid process [32]. Os Odontoideum has association with clinical entities like Down’s syndrome. Instability can be demonstrated as movement by 8 mm or more with respect to C2, as seen in flexion extension view [2]. Treatment includes atlanto-axial arthrodesis. Methods of fixation include C1-C2 transarticular screws and C1 lateral mass screws with C2 pedicle or trans-laminar screws. In case of incompetent, assimilated or very thin C1 arch, Occipito Cervical Fusion is the option [33].

### Hangman’s Fractures

It is commonly seen in age less than 2 years caused by hyperxextension injury. Roentgenograms show subluxation of C2 over C3 and lysis in pars of C2. This is commonly misinterpreted as pseudosubluxation or persistent synchondrosis or congenital spondylolysis [34]. Halo immobilization is advised for a period of 8-12 weeks. In cases of nonunion or instability anterior C2-3 arthrodesis can be performed [35].

### Lower Cervical Spine Injuries

Vertebral body injuries are more common in older children and occur through cartilaginous endplate. These are unstable and can cause neurologic injury. Treatment includes closed reduction and immobilization in halo cast [36]. Other injuries are similar to that seen in adults. Compression fractures are most common injuries seen in lower cervical spine followed by facet dislocations. Compression fractures are stable and should not be mistaken with normal wedging of cervical vertebras. It is usually treated with halo immobilization. If there is neurodeficit or retropulsed fragment then decompression with arthrodesis can be performed. Facet dislocations can be unilateral affecting the nerve root or bilateral affecting the spinal cord. They are primarily reduced by closed methods, by means of traction. If closed method fails, open reduction can be performed [2].

### Spinal Cord Injury Without Radiographic Abnormality (SCIWORA)

The term was coined by Pang and Wilberger in 1982, for condition involving spinal cord injury without any evidence of fracture or dislocation on plain roentgenograms and CT scan.

With advent of MRI, the term has now changed to spinal cord injury without neuroimaging abnormality [37]. SCIWORA is seen commonly in children and upper cervical spine is commonly affected. MRI can demonstrate cord edema, cord hemorrhage or ischemia. Treatment includes immobilization for 12 weeks followed by dynamic radiographs to detect instability [38].

## CONCLUSION

Spinal injuries in children are rare and typical. They are often missed and can have fatal consequences. Thorough understanding of pediatric anatomy and injury patterns helps in early diagnosis. Systematic approach towards patients helps to prevent morbidity and mortality.

## Figures and Tables

**Fig. (1):**
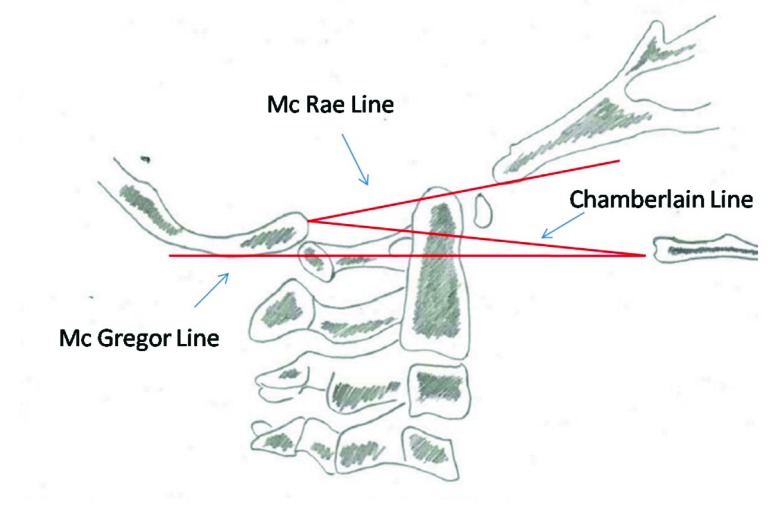
Common lines used to assess basilar invagination.
